# Reading Mendelian randomisation studies: a guide, glossary, and checklist for clinicians

**DOI:** 10.1136/bmj.k601

**Published:** 2018-07-12

**Authors:** Neil M Davies, Michael V Holmes, George Davey Smith

**Affiliations:** 1 Medical Research Council Integrative Epidemiology Unit, University of Bristol, BS8 2BN, UK; 2 Population Health Sciences, Bristol Medical School, University of Bristol, Barley House, Oakfield Grove, Bristol, BS8 2BN, UK; 3 Medical Research Council Population Health Research Unit, University of Oxford, UK; 4 Clinical Trial Service Unit and Epidemiological Studies Unit (CTSU), Nuffield Department of Population Health, University of Oxford, Richard Doll Building, Old Road Campus, Roosevelt Drive, Oxford OX3 7LF, UK.; 5 National Institute for Health Research Oxford Biomedical Research Centre, Oxford University Hospital, Oxford, UK; 6 National Institute for Health Research Bristol Biomedical Research Centre, Oakfield House, Oakfield Grove, Bristol BS8 2BN, UK

## Abstract

Mendelian randomisation uses genetic variation as a natural experiment to investigate the causal relations between potentially modifiable risk factors and health outcomes in observational data. As with all epidemiological approaches, findings from Mendelian randomisation studies depend on specific assumptions. We provide explanations of the information typically reported in Mendelian randomisation studies that can be used to assess the plausibility of these assumptions and guidance on how to interpret findings from Mendelian randomisation studies in the context of other sources of evidence

**Summary points**

Mendelian randomisation is a research method that provides evidence about putative causal relations between modifiable risk factors and disease, using genetic variants as natural experiments
Mendelian randomisation is less likely to be affected by confounding or reverse causation than conventional observational studies
Like all analytical approaches, however, Mendelian randomisation depends on assumptions, and the plausibility of these assumptions must be assessed
Moreover, the relevance of the results for clinical decisions should be interpreted in light of other sources of evidence
We provide a critical appraisal checklist that can be used to assess and interpret Mendelian randomisation studies

Understanding whether a biomarker or behaviour causes ill health is central to evidence based medicine, drug development, and better informed clinical decision making. Ideally, evidence of causal effects comes from well conducted randomised trials. Clinicians are well versed in the strengths and limitations of such trials and have an increasingly sophisticated understanding of traditional analyses of observational studies. But they may be less aware of the strengths and limitations of a more recently developed approach to analysing observational data known as Mendelian randomisation. Although numerous guides exist for conducting^1^ ^2^ ^3^ and reporting Mendelian randomisation studies and related methods,^4^ ^5^ here we focus on helping clinicians and practitioners read and interpret them. Our goal is to provide explanations of core concepts and recent developments in Mendelian randomisation methods.

## A method to overcome confounding

Mendelian randomisation is an analytical method that uses genetic variants as instrumental variables for modifiable risk factors that affect population health.^1^ ^6^ ^7^ ^8^ It is increasingly being used because it can overcome a major limitation of evidence from observational studies: unmeasured confounding.^6^ ^9^ Suppose, for example, we wanted to investigate the effects of alcohol consumption on blood pressure with a view to understanding the overall relationship of alcohol with risk of coronary heart disease. One source of evidence is the association between alcohol and blood pressure in observational studies. This association may be a poor indicator of the causal effects of alcohol if there are other factors—“confounders”—that influence both alcohol intake and blood pressure. Many epidemiological methods attempt to correct for, or minimise, observed differences in confounders between study participants. These methods can give useful evidence about causal relations if we measure enough confounders so that, after adjustment or matching, study participants who consume different amounts of alcohol are otherwise comparable. But this assumption is unverifiable; if it does not hold, then findings from observational studies will be biasedestimates of causal effects. 

People who consume more alcohol may also have other risk factors for cardiovascular disease, such as smoking more heavily than those with lower alcohol consumption. The confounding factor (smoking) induces a positive association between the risk factor (alcohol) and an outcome (blood pressure); interpreting this as causal would be misleading. Measuring a confounder does not perfectly characterise it, so measurement error leads to residual confounding, even after apparent statistical adjustment. Reverse causality is a form of confounding that is difficult to account for. It arises if the outcome or preclinical aspects of the disease that lead to the outcome affect the risk factor. People with symptoms of cardiovascular disease, for example, may consume less alcohol than those without symptoms. This would lead to a negative association between a risk factor (alcohol) and an outcome (cardiovascular disease); interpreting this as being because alcohol consumption decreases the risk of cardiovascular disease would be misleading.

Mendelian randomisation uses genetic variants, which are fixed at conception, to support causal inferences about the effects of modifiable risk factors, which can overcome some types of confounding. In the case of alcohol and blood pressure, a variant in the *ALDH2* gene (specifically the minor A allele of rs671, rather than the wild type or major allele G) found in east Asian populations slows the metabolism of acetaldehyde, which causes a flush response and other adverse responses to alcohol consumption. In a study of 4057 people selected from the general population, 170 of 1919 men carried two copies of the A allele and drank an average of 1.1 g of alcohol a day, whereas those with no copies drank 23.7 g.^10^ ^11^ If men with one or more copies of the A allele have lower blood pressure, then this implies that lower alcohol consumption decreases blood pressure.^11^ But this inference relies on several assumptions. The two key challenges when reading Mendelian randomisation studies are evaluating the plausibility of the underlying assumptions and interpreting the results. We discuss these challenges below, using terms defined in the glossary (box 1).

Box 1 **Glossary of common terms used in Mendelian randomisation studies**

Readers interested in more detailed discussions of these terms are encouraged to read the references.^1^ ^9^ ^12^ ^13^

**Concepts**

*Instrumental variables*—variables that are associated with the risk factor of interest, that are not related to confounders, and that affect the outcome only through the risk factor.^14^ An instrumental variable can be any trait (not necessarily a genetic variant) that meets these criteria, but the nature of genetic inheritance means that genetic variants are often plausible instrumental variables
*Mendelian randomisation*—the use of genetic variants as instrumental variables to investigate the effects of modifiable risk factors for disease^6^ ^15^
*Multiple instruments*—the use of more than one genetic variant in a Mendelian randomisation analysis
*Allele score*—the number of alleles associated with an increase in the risk factor of interest. These genetic variants are normally identified in large genome-wide association studies. The statistical efficiency (power) of allele scores can be increased by weighting each variant by the size of its association with the risk factor^16^
*Weak instrument bias*—can occur in Mendelian randomisation studies when using one or more genetic variants that only explain a small proportion of the variation in the risk factor, coupled with a small sample size
*Pleiotropic effects*—the effects of a genetic variant on multiple biological pathways. These can either affect the outcome through another trait or pathway to the one under investigation, known as horizontal pleiotropy, or affect other traits through the risk factor of interest, known as vertical pleiotropy.^1^ Horizontal pleiotropy is a violation of the instrumental variable assumptions because the effects of the genetic variant on the outcome are not exclusively through the risk factor; this is problematic for Mendelian randomisation studies. Vertical pleiotropy is in fact the essence of Mendelian randomisation—showing that one factor influences a downstream outcome—and is in general not problematic^1^

**Statistical methods**

*Single sample Mendelian randomisation*—using one dataset in the instrumental variable analysis to yield the causal estimate of the risk factor on the outcome
*Two sample Mendelian randomisation*—using two different study samples to estimate the instrument-risk factor and instrument-outcome associations to estimate a causal effect of the risk factor on the outcome. This can be useful when the risk factor or outcome, or both, are expensive to measure.^17^ ^18^ It also provides an opportunity to substantially increase the statistical power, by incorporating data from multiple sources, including large consortia^17^
*MR Egger regression*—a statistical technique that allows one or more genetic variants to have pleiotropic effects, as long as the size of these pleiotropic effects is independent of the size of the genetic variants’ effects on the risk factor of interest^19^

**Properties of the genetic instrument**

*Testing between observational and Mendelian randomisation estimate*—tests include the Hausman test (for continuous outcomes in single sample Mendelian randomisation) and tests for difference in estimates (for binary outcomes in single sample and two sample Mendelian randomisation)^20^ ^21^
*Tests of instrument strength*—to evaluate the strength of the association between the instrument (the genetic variant) and the risk factor (for example, the partial F statistic and R^2^)^22^

## What assumptions does Mendelian randomisation depend on?

**Fig 1:**
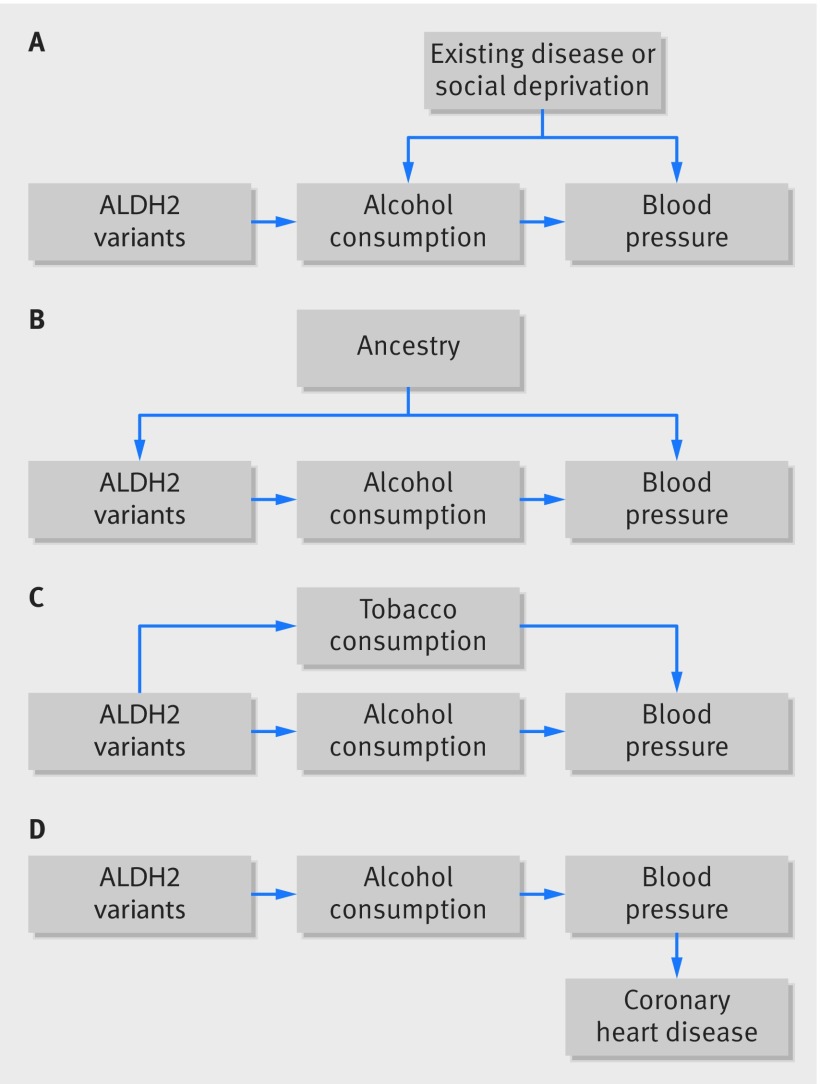
Examples of Mendelian randomisation and potential violations of assumptions. (A) A simplified causal diagram depicting confounding of the association of alcohol consumption and blood pressure by existing disease or social deprivation. The instrumental variable assumptions are that the genetic variants are associated with the risk factor, that theyhave no other influence on the outcome, except through alcohol, and that there are no confounders of the genetic variants-outcome association. (B) Confounding by ancestry could occur if variants associated with alcohol consumption had different frequencies in different ethnic groups in the population sampled and if cultural differences affected blood pressure between ethnic groups. This would violate the second instrumental variable assumption— the independence assumption. (C) An example of horizontal pleiotropy, in which the genetic variants associated with alcohol consumption also affect tobacco consumption (violating the third assumption— the exclusion restriction assumption). (D) An example of vertical pleiotropy, in which the effect of *ALDH2* on coronary heart disease is mediated by blood pressure. This example does not violate the Mendelian randomisation assumptions and does not cause bias.

Valid instrumental variables are defined by three key assumptions (table 1, fig 1): that they associate with the risk factor of interest (the relevance assumption); that they share no common cause with the outcome (the independence assumption); and that they do not affect the outcome except through the risk factor (the exclusion restriction assumption). A single genetic variant could plausibly meet these conditions if the biological process linking the variant with the risk factor is well understood. But in many cases Mendelian randomisation studies include multiple genetic variants, which can be used in sensitivity analyses to evaluate the underlying assumptions. Generally, the three key assumptions must hold for each of the genetic variants. We describe common strategies for assessing the plausibility of these assumptions and give examples, where possible, from published studies.

**Table 1:** Three key assumptions that must hold for a Mendelian randomisation study to be valid

Assumption	Description	Tools to assess plausibility	
		Single sample	Two sample
Relevance assumption	The genetic variants associate with the risk factor of interest	The partial F statistic and partial r squared, or risk difference	Variants are associated with the risk factor in a large genome-wide study
Independence assumption	There are no unmeasured confounders of the associations between genetic variants and outcome	Covariate balance tests and bias component plots. Adjusting for principal components of population stratification	Evidence from large genome-wide association studies on the association of the genetic variants used as instruments with other baseline covariates
Exclusion restriction	The genetic variants affect the outcome only through their effect on the risk factor of interest	Biological knowledge, tests of association of the genetic variants and potential alternative mediating pathways	Evidence from large genome-wide association studies that the genetic variants associate with alternative pathways. MR Egger test for pleiotropy, Cook’s distance evaluation of outliers

### Sources of data in Mendelian randomisation

Historically, a typical Mendelian randomisation study required measures of genotypes (variants in *ALDH2* gene), risk factor (alcohol consumption), and outcome (blood pressure) from the same sample of people. This approach is known as single sample Mendelian randomisation. Two sample Mendelian randomisation involves two separate study populations; for example, data on the *ALDH2* genotype and alcohol consumption are measured in one sample, and *ALDH2* genotype and blood pressure in the other.^17^ ^23^ This design has two advantages. Firstly, neither the risk factor nor the outcome needs to be measured in all studies, which is particularly useful if they are difficult or expensive to measure. Secondly, it allows the summary results from genome-wide association studies to be used, which can be very large (often >50 000) and thus highly precise (table 2 and table 3).

**Table 2:** Publicly available data sources for two sample Mendelian randomisation studies

Consortium name	Description	Most recent sample size
BCAC^24^	Breast cancer	256 123
CARDIoGRAMplusC4D^25^	Coronary artery disease and myocardial infarction	184 305
CKDGen^26^	Chronic kidney disease	111 666
DIAGRAM^27^	Diabetes	159 208
EAGLE^28^	Antenatal and early life and childhood phenotypes	47 541
EGG^29^	Early growth	153 781
GIANT^30^	Height, BMI, and other adiposity traits	693 529
GLGC^31^	Global lipids genetics consortium	331 368
ISGC^32^	Stroke	84 961
MAGIC^33^	Glucose and insulin related traits	224 459
PGC^34^ ^35^	Psychiatric genetics, alcohol and tobacco, and other related traits	>500 000
SSGAC^36^	Educational attainment and wellbeing	293 723

**Table 3:** Databases of genome-wide association study results

Data source	Description	Number of traits	Integrated with statistics package?
MR-Base	A curated database of genome-wide association study results with integrated R package for MR^23^	Over 1000	Yes
PhenoScanner	A curated database of genome-wide association study results with integrated R package for MR^37^	Over 500	Yes
GWAS catalog	Searchable database of genome-wide association study results^38^	Over 24 000	No

Statistical power is usually much higher in two sample studies. These advantages come with two additional assumptions: the two samples are assumed to represent the same underlying population, and overlap in participants between the two samples can cause bias towards the risk factor-outcome association.^17^ ^39^

### Single or multiple genetic variants

The simplest applications of Mendelian randomisation use a single genetic variant as an instrument for the risk factor. Some of the most persuasive published studies use a single genetic variant with a relatively well understood function, so the core assumptions can be supported by biological knowledge. One such example is the use of a missense variant in the *PCSK9* gene, which modifies the function of proprotein convertase subtilisin/kexin type 9, an enzyme that degrades the low density lipoprotein (LDL) receptor on hepatocytes. *PCSK9* variants are associated with altered blood concentrations of LDL cholesterol and risk of heart disease.^40^ ^41^ This provides further evidence that LDL cholesterol causes heart disease, and also indicates that drugs that inhibit PCSK9 may have cardiovascular benefits. This was confirmed in phase III clinical trials.^42^ ^43^

In most circumstances, however, single genetic variants individually typically explain only a very small proportion of the variation in a phenotype; investigators may refer to these as “weak instruments,” particularly in modest sample sizes. Studies using variants with modest effects in small samples are likely to have very low statistical power and can be biased. To overcome this, investigators have developed methods that use multiple genetic variants that collectively explain more of the variation in a risk factor than a single variant and thus have more statistical power. One way of using multiple genetic variants is to use single nucleotide polymorphisms (SNPs, the most common form of DNA variation among people) as individual instruments in a statistical regression model in the setting of a single or two sample dataset (multiple instruments approach). The second way is to aggregate the variants into an allele score (also called a genetic risk score, gene score, or a multilocus allele score).^16^ The allele score is effectively a single instrumental variable that can be used to predict the risk factor in a Mendelian randomisation analysis.

Genetic variants in these scores are often weighted by their associations with the risk factor to maximise statistical power. Many recent Mendelian randomisation investigations of complex traits (such as blood pressure, BMI, or blood lipids)^44^ have used multiple variants because they can help identify pleiotropy or other violations of the underlying assumptions.

### Genetic pleiotropy

**Fig 2:**
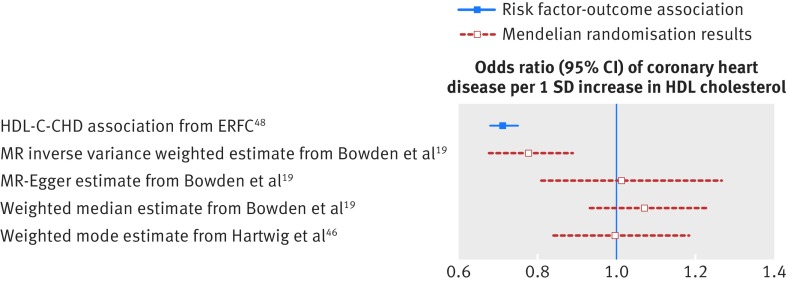
Example of genetic pleiotropy in Mendelian randomisation: HDL cholesterol and risk of heart disease. Variants associated with HDL cholesterol are likely to have pleiotropic effects on risk of heart disease because they also associate with LDL cholesterol and triglycerides.^2^ ^45^ Thus the inverse variance weighted Mendelian randomisation estimate, which assumes no pleiotropy, provides (biased) evidence of a protective role for HDL cholesterol in coronary heart disease. But the estimates using MR Egger, weighted median, and weighted mode, which allow for genetic pleiotropy, are attenuated towards the null. The MR Egger estimator assumes that for the variants with pleiotropic effects on coronary heart disease the magnitude of these effects do not correlate with the magnitude of the variants’ effects on HDL cholesterol. These results suggest that the inverse variance weighted estimate is driven by genetic pleiotropy^19^ ^46^ ^47^ ^48^ and that HDL cholesterol is unlikely to have a major causal role in the development of coronary heart disease. CHD=coronary heart disease; ERFC=Emerging Risk Factors Collaboration; HDL-C=high density lipoprotein cholesterol; SD=standard deviation

Genetic variants may affect the outcome through pathways other than through the risk factor of interest (so called horizontal pleiotropic effects, fig 1C). Genetic variants associated with alcohol consumption, for example, may affect other behaviours such as smoking, which would invalidate the exclusion restriction. When using single or multiple genetic variants, Mendelian randomisation estimates require that genetic variants do not have such horizontally pleiotropic effects.^16^ This means the results could be biased if a genetic variant or an allele score has pleiotropic effects on the outcome that are not mediated through the risk factor of interest (fig 2). Genetic variants can also affect the outcome through a pathway affected by the risk factor of interest (vertical pleiotropic effects, fig 1D). This does not invalidate the instrumental variable assumptions and does not result in bias.

Various methods have been developed that allow for genetic pleiotropy.^19^ ^46^ ^47^ These provide useful sensitivity analyses to explore whether a finding depends on the assumption that all the variants have no pleiotropic effects. One such approach, known as the median estimator, can provide reliable evidence as long as at least half the genetic variants have no pleiotropic effects.^47^ ^49^ A second method, known as MR Egger regression, allows all variants to have pleiotropic effects, provided they are not proportional to the variants’ effects on the risk factor of interest.^19^ A comprehensive analysis of cross trait genetic effects indicates that the magnitude of genome-wide associations are generally independent across traits that are not causally related, so the assumption may be plausible for many potential relationships.^50^ MR Egger regression yields less precise estimates than other methods, owing to a power penalty. Most of these methods assume that the risk factor has the same effect on everyone.

## How to assess the key assumptions

### Tests of instrument strength—the relevance assumption

The power of a Mendelian randomisation study is determined by sample size and strength of the association between the proposed instrument and risk factor. Weak instruments that poorly predict the risk factor cause three problems.^12^ Firstly, they provide very little statistical power to test hypotheses. Secondly, bias due to violations of the core instrumental variable assumptions, such as horizontally pleiotropic effects of variants, will be amplified. Thirdly, even when using very large samples, results using weak instruments are biased towards the outcome-risk factor association in the single sample setting and towards the null in the two sample setting. Precision (assessed with confidence intervals) is underestimated. Weak instruments can be detected using the F statistic for single sample settings.^51^ A rule of thumb is that the F statistic should be greater than 10. Exceeding this threshold indicates that a result based on a valid instrumental variable ought not to suffer substantially from weak instrument bias but does not guarantee sufficient statistical power to test a specific hypothesis.

### Independence and exclusion restriction assumptions

Confounding by violations of the independence assumption (no confounders) and exclusion restriction (genetic instruments work only through the risk factor) can be investigated by estimating the relation between the genetic instrument and a wide range of characteristics, analogous to the balance of baseline characteristics between treatment arms in a randomised trial.^52^ ^53^ ^54^ Although these assessments cannot prove that the independence and exclusion restriction assumptions hold, they can provide evidence regarding their (lack of) plausibility.

One promising way to evaluate the plausibility of the assumptions is using “negative control” populations. Genetic variants that are known to affect alcohol consumption, for example, should not be associated with outcomes in populations that rarely or never drink, such as children or women in some societies.^11^ This tests the exclusion restriction and independence assumptions. If investigators found an association between *ALDH2* variants and blood pressure in a non-drinking population, the instrumental variable assumptions may not hold in the original population either. The usefulness of negative controls depends on them being well reasoned and, of course, available.

## How are Mendelian randomisation studies analysed?

The simplest approach is to report the association of the genetic variants and the outcome. This test is relatively robust and should be reported in all studies. It provides evidence as to whether the risk factor causes the outcome, but it is not informative about the size of the effect. The magnitude of the causal effect can be estimated by dividing the genetic variant-outcome association by the genetic variant-risk factor association. This ratio is known as the instrumental variable or Wald estimate.^55^

### Inclusion of, or stratifying by, the risk factor of interest

To assess whether a genetic variant (or several in combination) mediates its effect on an outcome through the primary risk factor (and thus to test for exclusion restriction), some studies adjust or stratify for the primary risk factor of interest.^56^ But this is problematic for several reasons. Firstly, a residual association of the genetic instrument with the disease after adjustment for the risk factor does not imply that the variant affects the disease through other pathways. The measured risk factor almost certainly does not completely account for the lifetime variation in the trait instrumented by the gene. A residual effect of the gene (on the outcome after adjusting for the measured risk factor) might therefore not indicate a violation of the exclusion restriction assumption. Secondly, adjusting or stratifying for the risk factor can lead to collider bias (see supplementary figure 1), in the same way that conducting a subgroup analysis by “on-treatment” response breaks the randomisation in a trial.^57^ To see why, consider a study investigating the effect of statins on coronary heart disease.^58^ The on-treatment (or “achieved”) LDL cholesterol concentration will break the randomisation and is likely to be biased by other characteristics that influence lipid levels (such as age, sex, diet, physical activity, and so on). Although this is a seemingly common retrospective approach to try and elucidate dose-response relatonships in a randomised trial, such analyses can be as vulnerable to confounding as observational analyses. Similarly, adjusting (or stratifying) a Mendelian randomisation analysis for the risk factor can induce spurious correlations between the genetic variants and the outcome.

### Why do some Mendelian randomisation studies adjust for other traits?

If a genetic variant is a valid instrument, inclusion of other covariates is not necessary, but they can increase statistical efficiency. Some Mendelian randomisation studies adjust for other characteristics, including directly measured traits (such as age, sex, or smoking) or associations between the genetic instrument and traits other than the risk factor of interest. The genetic variant-risk factor and genetic variant-outcome associations should be adjusted for the same covariates. If not, the instrumental variable estimate may be biased. For example, if a two sample Mendelian randomisation study of the effect of BMI on blood pressure used a blood pressure genome-wide association study that adjusted for BMI, then the estimate would be unreliable and the direction of effect could even be reversed. These problems are an area of active methodological research.

In some samples, the association between a genetic variant and outcome may be confounded by hidden population structure. This can be tackled by adjusting for genetic ancestry or restricting to ethnically homogenous samples. Suppose a study sampled data from an ethnically mixed east Asian and European population. The minor allele of the alcohol variant rs671 is extremely rare in European populations, so an association between this variant and an outcome in this sample could be due to differences in ethnicity (fig 1B). This problem could be mitigated by stratifying by ethnicity or adjusting for genetic principal components (which are calculated in datasets with genome-wide arrays and provide proxy measures of genetic ancestry). The UK Biobank, for example, provides up to 40 principal components for adjusting genetic analyses for differences in ethnicity. This improves reliability, as has been done in a recent Mendelian randomisation study of adiposity and risk of heart disease and diabetes.^59^ If a study depends on these adjustments—that is, there are major differences between the unadjusted and adjusted findings—the results should be treated with caution. At the very least the source of potential bias must be investigated and justified, just as for residual confounding in a conventional epidemiology study.

### Why do some Mendelian randomisation studies remove variants from the genetic instrument?

Some studies manually remove (or “prune”) genetic variants thought to be pleiotropic from a genetic instrument to provide a more reliable estimate of the association. Voight and colleagues used genetic variants that were exclusively associated with HDL cholesterol (not LDL cholesterol or triglycerides) to investigate the relationship between HDL cholesterol and heart disease.^60^ Including or excluding a genetic variant in this way can be arbitrary and limited by available data; such pruning should be done with caution (if at all) and for well justified and transparent reasons. Furthermore, as the sample size increases, genetic variants will be identified as showing associations with multiple traits owing to vertical or horizontal pleiotropy.^61^ Excluding variants on the basis of vertical pleiotropic effects will bias findings, since these effects would be ones seen when manipulating the risk factor of interest.^62^ One or more variants might be excluded from sensitivity analyses if they are obvious outliers based on visual assessment of the data or using more formal approaches such as Cook’s distance.^63^

## How are Mendelian randomisation studies reported?

Having checked whether the assumptions underpinning a Mendelian randomisation analysis are valid (table 1), readers should be familiar with how such studies are reported (box 2). They may report the association of one or more genetic variants with the outcome or provide an estimate of the causal (instrumental variable) effects of the risk factor on the outcome, or both. Ference and colleagues report the association between SNPs that alter LDL cholesterol and risk of heart disease, showing compelling genetic evidence of a dose-response association across multiple independent genetic loci. These SNPs can also be used to provide an “overall” causal effect of LDL cholesterol on the risk of coronary heart disease (odds ratio 0.46, 95% confidence ratio 0.41 to 0.51 per 1mmol/L lower LDL cholesterol).^64^ These associations leverage genetic differences that occur at conception and can detect whether differences in the risk factor at any point over the life course affect the outcome. As a guide to future research and drug development, it is important to remember that findings of Mendelian randomisation potentially reflect lifetime differences in risk factors.^65^ ^66^

Box 2 **Critical appraisal checklist for evaluating Mendelian randomisation studies**

Some key questions readers can ask below.

**Core Mendelian randomisation assumptions**

Is there sufficient evidence that the genetic variants are robustly associated with the risk factor of interest?
Are the genetic variants associated with potential confounders? Do the authors present this relationship?
Is there any way for the genetic variants to affect the outcome through alternative pathways (horizontal pleiotropy)? Do the authors present alternative Mendelian randomisation approaches (such as MR Egger, median, and mode estimators, or use of “negative control” populations) to investigate this more fully?

**Methods reporting**

**All studies**

Are the effect and other alleles coded in the same direction for the exposure and outcome?

**Two sample studies**

Were the two samples drawn from the same population?
Were the two samples independent?
Was the analysis restricted to independent variants (that is, pruned of SNPs in linkage disequilibrium) or did the analysis allow for the correlation between variants?

**Data presentation**

Do the authors present the results as a genetic association, an instrumental variable estimate, or both?
If they provide an instrumental variable estimate, do they compare it with the conventional observational estimate?
Do the authors provide sensitivity analyses such as MR Egger, weighted median, and mode Mendelian randomisation, or use negative control populations?
Do the authors manually pick and choose which SNPs go into the instrument to tackle pleiotropy? If so, is the approach and justification clear?
Do the authors provide the data that they used (especially for Mendelian randomisation analyses conducted at the summary level) in a supplement to allow researchers to reproduce their findings?

**Interpretation**

If the Mendelian randomisation estimate is similar to the observational estimate and provides evidence in support of a causal effect, could it be due to weak instrument bias in a single study or confounding through, for example, horizontal pleiotropy?
If the Mendelian randomisation estimate differs from the observational estimate and provides little evidence of a causal effect, could this be due to weak instrument bias when using two different samples or negative confounding due to pleiotropy?
Mendelian randomisation provides estimates of the effects of the risk factor over a lifetime, and the numerical effect estimates may not be clinically meaningful. Will interventions at a specific age have the same sized effects?
Are the 95% confidence intervals of the Mendelian randomisation estimate sufficiently precise to identify the observational estimate and a clinically meaningful difference?

**Clinical implications**

Do the results triangulate with other forms of evidence? Could a clinical trial be conducted to provide definitive evidence, as in the case of PCSK9 inhibitors?
If a randomised clinical trial is not feasible (such as in the case of alcohol consumption and risk of heart disease) or unlikely to be conducted in the short term (such as the case of lifestyle interventions to lower BMI and risk of heart disease), and there is existing evidence from multiple Mendelian randomisation studies and other robust study designs that converge on a similar result and show consistency of association, this information can be used to guide patient care; for example, advising weight loss to prevent heart disease or advising against moderate alcohol consumption to prevent cardiovascular disease

### Special cases of reporting findings

#### Testing for differences between Mendelian randomisation estimates from different SNPs

If multiple genetic variants and biological pathways influence the risk factor, we can test whether the effect of the risk factor on the outcome is similar when using different variants using Hansen tests or Cochrane’s Q test for individual level data and summary data, respectively.^67^ ^68^ If these test statistics are large (yielding correspondingly small P values), the estimated causal effects of the risk factor may vary across the population or between variants. This might be due to multiple pathways causing the outcome. If these tests cannot be rejected, sample size and statistical power may be insufficient to detect differences that do exist and, therefore, may be falsely reassuring.

#### Comparing findings from observational and Mendelian randomisation analyses in the same dataset

**Fig 3:**
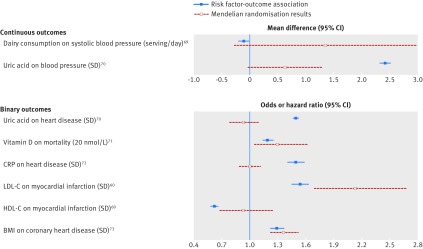
Example associations between risk factors and outcomes from traditional observational epidemiology and Mendelian randomisation instrumental variable estimates. 60 69 70 71 72 73 For some associations—such as vitamin D and mortality—the Mendelian randomisation results potentially confirm some causal relation. For other associations—CRP and heart disease—the Mendelian randomisation results are consistent with there being no causal effect. BMI=body mass index; CRP=C reactive protein; HDL-C=high density lipoprotein cholesterol; LDL-C=low density lipoprotein cholesterol

For continuous outcomes in single sample analyses, the Hausman test can be used to assess whether the Mendelian randomisation and linear regression results (obtained from the same dataset) are systematically different.^20^ Differences could occur because the linear regression results are biased by residual confounding, the independence or exclusion restriction assumptions underlying the instrumental variable regression approach are invalid, or both. Finally, the effects being estimated by the two methods may not be the same—the Mendelian randomisation estimate reflects the effects of lifelong perturbations in the risk factor, whereas linear regression results may reflect more acute effects. Mendelian randomisation estimates are almost always less precise and have wider confidence intervals than linear regression (fig 3), so tests for difference often have low statistical power.^74^

## How should findings from Mendelian randomisation be interpreted?

**Fig 4:**
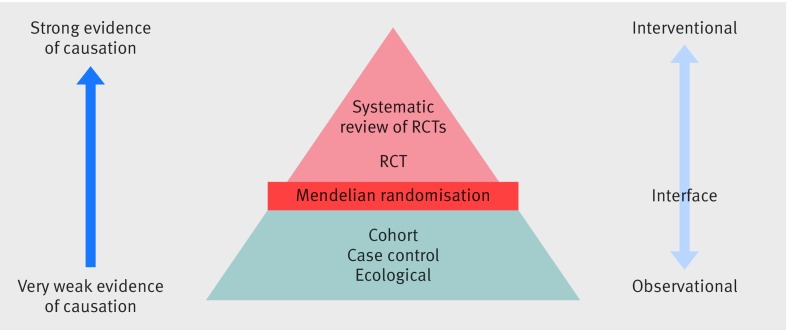
A hierarchy of observational and experimental data. Mendelian randomisation studies sit at the interface of experimental and observational studies. Their findings can be used to provide more reliable evidence to guide interventional research and provide information about potential public health interventions when a randomised controlled trial may not be feasible. Although we adapt the conventional pyramid of evidence for presentation purposes, we consider that triangulation of findings from different study designs should be used.^75^

The findings from Mendelian randomisation studies, which are less susceptible to confounding and reverse causality bias, sit at the interface between traditional observational epidemiology and interventional trials (fig 4). A well conducted Mendelian randomisation study that reasonably satisfies the above assumptions often provides more reliable evidence than a conventional observational study. But the findings should be interpreted in the context of existing evidence from other sources, using different study designs,^75^ and clinical guidelines should not be rewritten solely on the basis of Mendelian randomisation results.

## Triangulating the evidence

Some studies report the Mendelian randomisation estimate in the context of traditional observational epidemiology (fig 3; supplementary table 1). When there is clear evidence that the associations differ, the Mendelian randomisation estimate can provide strong evidence that the traditional observational estimate arises from confounding and/or reverse causality. In the case of C-reactive protein and coronary heart disease, the Mendelian randomisation estimates suggest that targeting CRP is unlikely to be a viable therapeutic target for the prevention of coronary heart disease.^72^ In contrast, when the Mendelian randomisation estimate has very wide confidence intervals that overlap the observational estimate and include the null (as in the case of dairy consumption and blood pressure),^69^ very little can be inferred from the Mendelian randomisation results.

Summary estimates from observational and Mendelian randomisation studies can be compared formally using tests for heterogeneity, such as Cochrane’s Q test and tests for differences in estimates.^21^ This can provide statistical evidence of a difference, but whether this should be interpreted as clinically meaningful is further outlined below.

### Clinical and public health implications

Clinical and public health decisions about potential interventions ideally require evidence about the size and direction of an effect for a specific population. We might want to know, for example, how reducing alcohol consumption by an average of one serving a day beginning at age 45 affects blood pressure five years later. But this must be approached with care because genetic variants generally relate to lifelong differences in a risk factor, not the effects at a specific time. *ALDH2* is associated with alcohol consumption from adolescence through middle age,^76^ so the Mendelian randomisation results (coupled with biological knowledge) indicate that consuming less alcohol would lead to lower blood pressure on average, but do not necessarily inform the effect size of an intervention at a specific time in life for a specific duration. Another example is the relation between vitamin D and multiple sclerosis, where data from Mendelian randomisation studies indicate a protective effect, but such protection may be limited to childhood and adolescence and not later adult life.^66^ ^77^ Biological and methodological knowledge about the relations between risk factors and outcomes is critical to interpreting Mendelian randomisation studies. Does risk accumulate over time? Does the risk factor have an acute effect? Is there a particular time in life where this risk factor has an effect on this disease?^6^ ^66^ ^78^ ^79^ This is why some studies focus on the association of the genetic variants and outcome,^56^ rather than providing a direct quantification of the causal effect scaled to a difference in the exposure of interest.

Consider another example, where the biology and context are important for the appraisal of Mendelian randomisation findings. Variants in the *CHRNA5* gene that associate with increased heaviness of smoking are (through smoking behaviour) associated with increased risk of lung cancer.^80^ People with these variants are also likely to find it more difficult to reduce smoking after diagnosis of lung cancer. But variants in this gene cannot be used to assess the effect of quitting or reducing smoking on mortality after diagnosis with lung cancer, because people with the risk variants will have smoked more heavily before diagnosis. The association between *CHRNA5* risk variants and mortality in people with lung cancer reflects an individual’s lifetime exposure to tobacco smoke, and we would not expect an instrumental variable estimate using *CHRNA5* to reflect the effect of current smoking on prognosis. Most importantly, the triggers of disease onset may be entirely different to the factors leading to progression.^81^ This means that Mendelian randomisation studies need to be performed using data from studies of disease progression and prognosis, rather than disease occurrence, to generate findings of direct relevance to treating disease.^81^ Finally, when an exposure is seemingly non-modifiable (such as height^82^ ^83^), a causal relation can point towards potential mechanisms or pathways that lead to disease (even if the actual risk factor under investigation is non-modifiable); such pathways are likely to be modifiable, providing new insights in disease aetiology.

## Summary

Mendelian randomisation studies can provide reliable evidence on the effect of modifiable risk factors for disease or ill health and can overcome some limitations of traditional observational epidemiology. In settings for which the instrumental variable assumptions are well justified (assessed as described above and using biological knowledge), the findings could help prioritise clinical trials or drug development and inform clinical or public health decision making.^84^ ^85^ ^86^

The conversation on how to conduct, report, and interpret Mendelian randomisation studies is still ongoing. Other areas of medical research, such as randomised controlled trials, have been greatly strengthened by close collaborations between methodologists, empirical researchers, and clinicians. Similar collaborations are needed to ensure the strengths and limitations of Mendelian randomisation are fully appreciated and realised.

## Web extra

Extra material supplied by authors

**Supplementary table:** Strength of genetic instruments

**Supplementary figure:** An illustration of collider bias

## Notes

Contributors and sources: All authors wrote and edit the manuscript, and approved the final draft. All authors confirm that they have contributed to this article and met the following three requirements: (a) they made significant contributions to the conception and design; (b) they drafted or revised the article for intellectual content; and (c) gave final approval of the published article. NMD is the guarantor.
