# Supplementary material for: Reading Mendelian randomisation studies: a guide, glossary, and checklist for clinicians
Source: BMJ. 2018 Jul 12;362:k601. doi: 10.1136/bmj.k601 (PMC6041728; doi:10.1136/bmj.k601)
Supplement: Supplementary file 1 — Supplementary figure: An illustration of collider bias [file davn040667.ww1.pdf]

Supplementary Table 1: Strength of genetic instruments, and examples of specification tests reported by the studies reported in Figure 2. All studies reported the strength of association between the variants and their risk factor of interest, most studies reported the association of the genetic variants and observed confounders, and most studies reported on the biological implausibility of pleiotropy.

				Assumption 1	Assumption 2	Assumption 3[b]
				Do the SNPs associate with risk factor?	Did the authors report on the association between SNPs and possible confounders?	Did the authors provide biological reasons why pleiotropy is unlikely?
Paper	Outcome	Risk Factor	Number of SNPs	Strength of association with phenotype F-statistic	Assessed association with confounders	The biological mechanisms of the SNPs are known
Ding et al.[52]	Systolic blood pressure (mmHg)	Dairy consumption (serving/day)	1	7.51a	Unclear	Yes
Palmer et al.[68]	Systolic blood pressure (mmHg)	Plasma uric acid (SD change)	1	1508	Yes	Yes
Palmer et al.[68]	Ischemic heart disease	Plasma uric acid (SD change)	1	1508	Yes	Yes
Afzal et al. [69]	All-cause mortality	Vitamin D (20 nmol/L)	4	327	Yes	Yes
C Reactive Protein Coronary Heart Disease Genetics Collaboration (CCGC)[51]	Coronary heart disease	C-reactive protein (SD change in ln(CRP))	4	239.8	Yes	Yes
Voight et al.[41]	Myocardial infarction	LDL cholesterol (SD change)	13	Yes, but no f-statistic reported	No	No
Voight et al.[41]	Myocardial infarction	HDL cholesterol (SD change)	14	Yes, but no f-statistic reported	No	No
Dale et al.[70]	Coronary heart disease	BMI	97	Yes, r-squares reported	No	No

Notes: [a] Z-statistic reported, 7.51. [b] Assumption 3 can be assessed either by biological knowledge or statistical tests. For example, variants in ALDH2 affect the metabolism of alcohol and are unlikely to affect blood pressure through pathways other than via alcohol consumption. Alternatively, if there are many SNPs that associate with the risk factor, then statistical tests such as MR-Egger can assess whether all the variants imply a similar size of effect on the outcomes. If many variants with different biological mechanisms imply similar sized effect, then pleiotropy is less likely. If estimates based on different variants provide different effect sizes, then this could be due to horizontal pleiotropy or could reflect multiple causal pathways from the same risk factor.

Supplementary figure 1: An illustration of collider bias (adapted from Gage and colleagues 2016)[71]

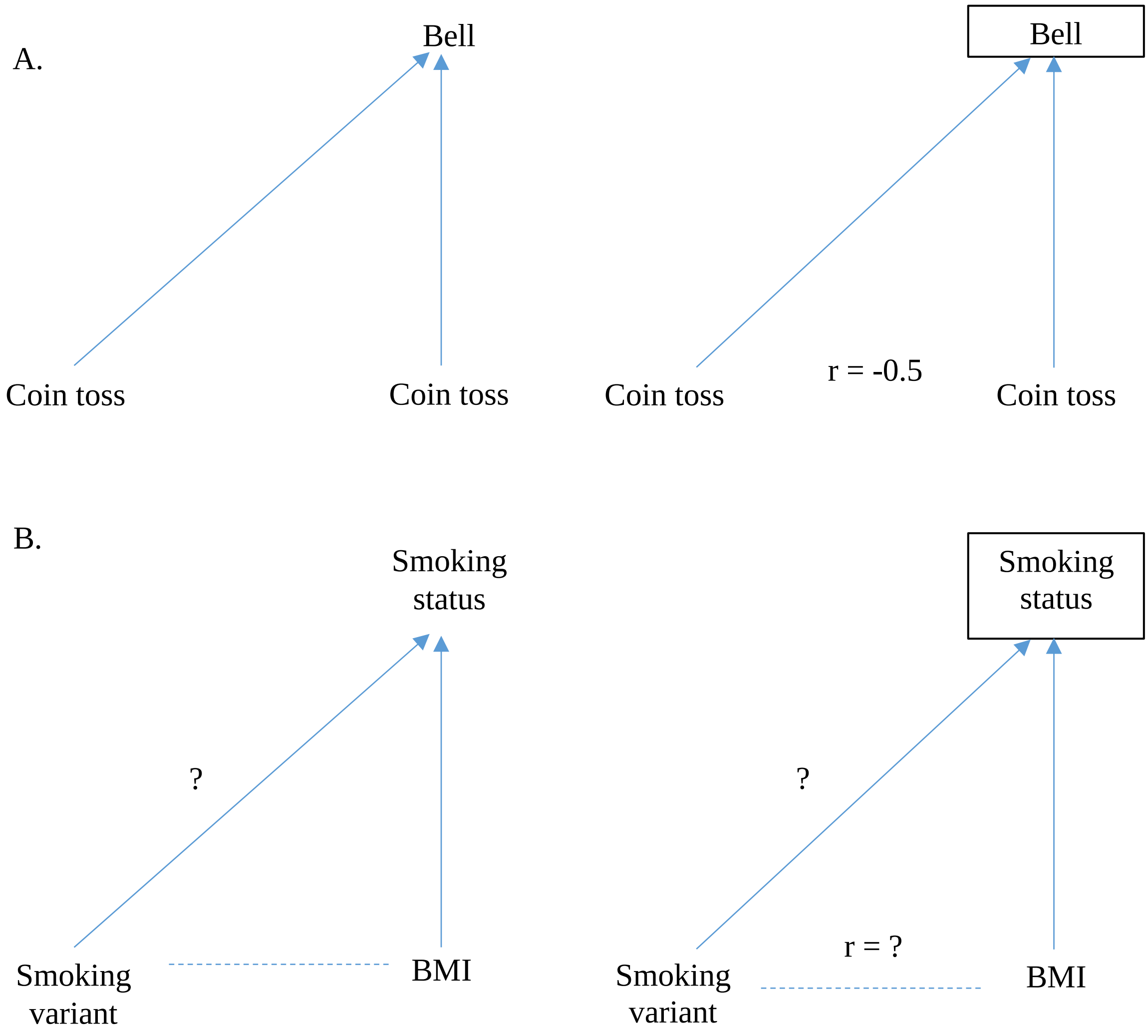

Panel A illustrates how collider bias can induce spurious associations. Two coins are tossed, if either coin is heads, then a bell is sounded. The result of each coin toss is random. However, if we stratify on “bell ringing”, then the results of the coin tosses will be negatively associated. This occurs because conditional on the bell ringing, on average two-thirds of the time one coin will be heads, and the other will be tails, so they will be negatively correlated. Panel B illustrates how collider bias could induce a spurious association between variants for smoking heaviness and BMI if both the smoking variants and BMI affected smoking status, and we stratify on smoking status. This bias can affect both observational and experimental studies.
